# Dynamics of factors associated with rates of COVID-19 cases and deaths in African countries

**DOI:** 10.1186/s12992-023-00918-9

**Published:** 2023-03-23

**Authors:** Iyabo O. Obasanjo, Zain Ahmad, Somasheker Akkaladevi, Adeyemi Adekoya, Olayide Abass

**Affiliations:** 1 Department of Kinesiology and Health Sciences, College of William and Mary, Williamsburg, VA 23185 USA; 2 College of William and Mary, Williamsburg, VA 23185 USA; 3 Reginald F. Lewis College of Business, Virginia State University, Petersburg, VA 23806 USA; 4 Bells University of Technology, Otta, Ogun State Nigeria

**Keywords:** Africa, COVID-19, Infectious Disease

## Abstract

**Background:**

African countries have not had the high case and death rates from COVID-19 as was predicted early in the pandemic. It is not well understood what factors modulated the rate of COVID-19 cases and death on the continent.

**Methods:**

We collated data from the World Bank data site, Our World in Data and Freedom House for African for 54 African countries who are members of the African Union. We used them as explanatory variables in two general linear model regression analyses. COVID cases and deaths per 100,000 obtained from WHO COVID-19 dashboard on August 12, 2021, as outcome variables in two prediction models.

**Results:**

GDP, percentage of population under 14 years of age, Maternal Mortality Ratio, number of international tourists visiting per year and public transportation closures were not significant in predicting COVID-19 cases. Higher percentage of unemployed adults in the population, lower percentage of the population over 25 years of age with secondary education, internal travel restrictions increased spread of COVID-19 while international travel restrictions were associated with lower COVID-19 cases per 100,000 population. Higher levels of democratization results in higher cases of COVID-19. Unemployment, education and democratization were still significant for COVID-19 death in the same direction as they were for COVID-19 cases. Number of tourism visitors per year was also associated with higher COVID-19 death rates but not with case rates.

**Conclusion:**

In African countries, internal movement restrictions enacted to inhibit COVID-19, had the opposite effect and enabled COVID-19 spread. Low Education levels and high unemployment were associated with having higher death rates from COVID-19. More studies are needed to understand the impact of tourism on COVID-19 and other infectious diseases arising from other regions on African countries, in order to put in place adequate control protocols.

## Key messages

- The main factors that predicted COVID-19 rates and deaths in the first year of the pandemic in African countries was high unemployment and low education rates.
- GDP per capita is not associated with COVID-19 rates in African countries but number of tourist visitors has a positive association.
- Restrictions in internal movements resulted in higher cases of COVID-19 but restrictions in international travel were effective in reducing transmission of the disease in African countries.

## Introduction

In the last week of December 2019, China confirmed the discovery of a new coronavirus infecting humans in Wuhan, Hubei Province as reported by the World Health Organization [34]. On March 11^th^ 2020, the World Health Organization (WHO) declared the outbreak of a pandemic, after cases started to occur in every continent and number of persons infected started to rise dramatically in the US and Europe [32]. Initial reports indicated that cases were spread by human to human transmission through droplets emanating from infected persons although the initial occurrence to infecting humans was from an animal source [34]. Subsequent reports hypothesized that transmission was higher indoors, and the disease was less severe in children [34]. While China’s response to the pandemic within its borders was judged to be exceptional by visiting WHO scientists, since Wuhan was a transportation hub with road, train and air transport connecting to the rest of China and air transport connecting globally, the opportunity for limiting global spread was probably lost in the early weeks of 2020 [34]. African countries started reporting cases in the Spring and Summer of 2020 and the initial cases for most African countries resulted from individuals who flew into the countries from Europe. Internal person-to-person infections soon started to occur with aggressive public health response from African governments. Such response included shutting down airports and borders and limiting internal human movements as well as instituting rigorous contact tracing and other attendant public health responses [13].

The ability of people living in poverty in Africa’s large cities to socially distance is virtually impossible, and the early expectation was that the disease would eventually overwhelm the existing and already fragile health systems of most countries on the continent [28]. Factors such as African countries being less connected in the movement of people to the rest of the world and therefore having fewer chances of introduction of a new disease from external sources, and swift actions by governments, were not taken into account in the early prediction of a dire situation in African countries from COVID-19. There is indication that the early awareness campaigns carried out by countries yielded benefits, with the populations becoming highly aware of the disease and how to prevent spread was beneficial [5]. It is clear that the expectation of COVID-19 devastating African countries has not occurred, and the reasons for this conundrum is not clear and has not been empirically established [13].

The image of Africa is that of a disease prone and disease-ridden continent due to the tropical, humid climate of much of the continent, but the fact is that most global pandemics that have been recorded have not factually and definitely been traced to an African source. The influenza pandemic of 1918 affected Africa but most likely originated in North America or in Asia [26]. It is relevant to point out that the HIV/AIDS pandemic was genetically traced to African primates but actually, the first human cases were found in San Francisco. While Ebola outbreaks are African in origin, however, but it has not so far resulted in a global pandemic leading to shutting down of almost all countries across the globe. Moreover, Ebola outbreaks have occurred in several African countries and the West African outbreak of 2014–2015 was the most spread-out scourge ever recorded, with several African having to carry out public health protocols of limiting human-movement internally and externally to curb spread of the disease. The difference with COVID-19 was that African countries were going to acquire COVID-19 cases from outside the continent and same procedures that were used to limit Ebola which was spread by intra-African travel may not limit COVID-19. In addition, the epidemiology and pathophysiology of COVID-19 are dissimilar to those of earlier pandemics since COVID-19 is acquired via airborne fomites, while Ebola is acquired by contact with any infected body exudation emanating from an infected person. Given that most of Africa is tropical with corresponding high rates of humidity which enables better survival of viral fomites and the fact that it has the collection of countries with the lowest GDP (Gross Domestic Product) per capita in the world coupled with weak health systems, the expectation is always for African countries to come out worse in infectious disease outbreaks without taking into account specific issues regarding each disease. The effect of introduction of a disease external to Africa to African countries based on the limited interconnections of Africa in terms of physical interactions of its people with the rest of the world has not been studied. The initial limited spread of COVID-19 in African countries could potentially be ascribed to the limited air travel connections African countries have with the rest of the world compared to the connections they have with each other which meant awareness campaigns and preparation for cases could occur ahead of the approaching pandemic.

While African countries have reportedly so far escaped having high rates of COVID-19 cases and death, as experienced by other regions of the world. It is however important to understand what factors were protective against having worse COVID-19 outbreaks, in order to understand how to ameliorate future pandemics especially for a disease that is being carried into the continent from other parts of the world [23]. Reviewing the factors that enabled African countries to weather the COVID-19 pandemic better than predictions anticipated is important, especially given the limited access to quality healthcare in most countries and the pervasive levels of poverty in many countries on the African continent. Understanding the factors that predict COVID-19 rates and death would provide information to help manage the next phase of the pandemic in African countries. The insights garnered would help advice countries on how to further manage the disease as it goes into a endemic phase globally, especially given the limited access to vaccines African countries are likely to encounter. Most importantly there is a need for empirical information on what factors mitigated the spread in African countries that will be useful for developing policy decisions and effective strategies for future highly contagious viral pandemics that might be introduced to the continent from other regions.

Now that there has been over a year of data accumulated on the pandemic and as it shifts from an epidemic stage to endemic stage, we can start to examine which factors contributed to or limited the spread of the disease on the African continent. In this paper we examine factors that influenced the rates of COVID-19 cases and deaths in African Countries during the first year of the pandemic. This study attempts to analyze COVID-19 occurrences with a view of establishing major predictors and the dynamics amongst them. It could also reveal how different mitigating strategies in COVID-19 were able to reduce spread of the disease in African countries.

## Methods

### Choice of variables

Higher GDP per capita of countries has been associated with higher rates of COVID-19 in several studies [7, 8]. The explanation given by Chang et al., 2020 was that higher GDP indicates high level of economic activity and international trade which would result in more human movement across the world that would lead to more opportunities for the disease to be picked up, introduced and spread within a country. African countries generally have low GDP per capita compared to other regions but there is enough internal variability to justify controlling for GDP in our prediction models. Since human movement into a country is the catalyst for initiation and spread of the disease we used tourism numbers into African countries in our model. Tourism numbers vary widely within African countries with few countries being high destination centers for tourism, and a majority of countries having low tourist visitor numbers compared to other regions of the world.

Several studies have found population age distribution to be an independent factor influencing rates of COVID-19 at country levels [8, 18, 30]. This is due to the virus generally causing more severe symptoms in older people who also tend to have more co-morbidities and more severe symptoms leads to more sneezing and other forms of extrusion of the virus which allows it to spread more [15, 19]. Countries with a higher proportion of younger people in the population as occurs in African countries compared to other regions will tend to have COVID-19 spread inhibited than in regions with older people making up a higher percentage of their population.

At the individual level, lower economic status is associated with worse outcome when infected with COVID-19 [27, 29]. We used unemployment levels to which tend to be high in African countries as a measure of poverty levels for the country. Education rates has been found to mitigate country level COVID-19 rates [7] and we therefore examined this in our study using percentage of the population with secondary education. To determine association of rate of COVID-19 mortality and morbidity with level of the healthcare system, Chang et al., 2020 used number of SARS cases, and number of hospital beds and found that having higher levels of both helped countries have less cases and death from COVID-19. African countries had almost no cases of SARS and the number of hospital beds reported for African countries tend to be inaccurately measured since oversight of private hospitals is not adequately carried out, so in order to measure country health system we used maternal mortality ratio which is a good measure of status of a health system to care for the sick and maintain the health of its population generally. Internal transportation restrictions, restrictions on movements and restrictions on international travel are all measures taken by governments to reduce the spread of COVID-19 although studies conducted using these variables have so far found no association with COVID-19 rates [7]. In this study, we use COVID-19 cases and death rates per 100,000 as the dependent variables in two different models. We included region as a distinct variable in each model to control for geographical and cultural differences with West Africa as the comparison region because it had the lowest case rate. GDP per capita was included in the model because we hypothesized that richer countries would have more external connections and more vulnerabilities for introduction of the virus. Democracies tend to be more effective in delivering services to citizens and public health is a public good that is expected that democratic norms would engender better management of COVID-19. However, Sorci et al. [30] and Chang et al. [7] found that higher levels of democratization were associated with worse COVID-19 case and death rates. In our model we examined how democratization influenced COVID-19 rates in African countries. COVID restrictions put in place by countries are very important in modulating COVID-19 spread and consequently, they were included in our models. Country level examination of COVID-19 restrictions were not significantly associated with COVID-19 rates in a study by Chaudhry et al. [8].

#### Sources of data

African Union membership list of countries [1], and the region that each country belongs (Central, Eastern, Northern, Southern and Western Africa) was used to create a database in Excel. Data on cumulative COVID-19 cases and death per 100,000 population for each country was obtained from the World Health Organization COVID-19 Dashboard [33] on 8/12/21. The explanatory variables, GDP per capita, Percentage of Population aged 0–14, % and Maternal Mortality Ratio, Tourism Visitor numbers, percentage of the population that finished Secondary education and unemployment rates for each country was obtained from the World Bank data site [35] for each country. We used the latest data available which was 2018 or 2019 for most countries. We obtained the democracy variable from Freedom House (Freedom [11] for 2020 and used the numerical score assigned to each country in our analyses rather than the ratings of free, partially free and not free. The higher the democracy score, the more democratic the country is. Both COVID data and explanatory variables were merged by country into the excel spreadsheet. Additional data on lockdown associated variables were obtained from the website Our World in Data website [25]. The 3 variables from the variables used from the website were Public Transportation Closures, Restrictions on internal movement, and International travel restrictions. Public Transportation Closures which was rated from 0 to 2: 0 was “No measures”, 1 was “Recommended closing (or reduce volume)”, and 2 was “Required closing (or prohibit most using it)”. Data on Restrictions on internal movement was rated from 0 to 2: 0 was “No measures”, 1 was “Recommend movement restriction”, and 2 was “Restrict movement”. Data on International travel restrictions was based on ratings from 0 to 4: 0 was “No measures”, 1 was “Screening”, 2 was “Quarantine from high-risk regions”, 3 was “Ban on high-risk regions”, and 4 was “Total border closure”. All Our World in Data Lockdown restriction variables for December 31^st^ 2020 were used, and represented changes made from the start of the pandemic until that date by governments as COVID-19 control measures.

#### The multiple regression model

Regression analysis was carried out using the General Linear Model (GLM) procedure in Statistical Analysis System (SAS) with COVID-19 cases per 100,000 and deaths per 100,000 as the outcome variables. There were 54 countries in the data set representing the 54 countries which are members of the African Union. Missing data for education and employment for some countries also lead to only 39 countries in the regression. We controlled for African Region in the model because African regions tend to have cultural similarities that would make countries in a region more or less prone to spread of specific diseases. For example, circumcision is almost universal across West Africa and the opposite is true of Southern Africa [16].

## Results

Table 1 has the descriptive statistics for all the variables and Table 2 has the correlation coefficients of the variables to each other. The overall model for predicting cases and death from COVID-19 was significant at *p* < 0.0001. Table 3 shows the regression results for COVID-19 cases and Table 4 those for COVID-19 deaths. There were regional differences in COVID-19 cases, with Central African region having significantly higher cases compared to Western Africa which was the comparison region. Central Africa currently has more countries with insecurity issues than other regions and that could account for the higher rate of cases in the region.

**Table 1:** Descriptive Statistics

Variable	Mean	Std. Deviation	N	Min	Max
COVID Deaths (cumulative total per 100,000 population)	20.55	36.73	54	0.08	179.55
COVID Cases (cumulative total per 100,000 population)	1284.80	2910.29	54	2.29	19,272.58
GDP per Capita in US Dollars (World Bank 2021 data)	2198.95	2278.90	54	274.01	11,425.10
Population ages 0–14 (% of total population)	38.91	6.78	54	16.78	49.67
Unemployment, total (% of total labor force) (modeled ILO estimate)	8.93	6.68	53	0.69	28.74
Educational attainment, at least completed upper secondary, population 25 + , total (%)	15.51	15.35	43	1.10	67.16
Maternal mortality ratio (modeled estimate, per 100,000 live births)	413.02	277.41	54	37	1150
International tourism, number of arrivals	1,848,558.00	3,342,396.71	50	30,000	14,797,000
Rating of Public transport closures from beginning of pandemic to Dec 31, 2020				0	2
Rating on Restrictions on internal movements from beginning of pandemic to Dec 31, 2020				0	2
Rating on International travel controls from beginning of pandemic to Dec 31, 2020				1	4
Freedom scores 2021 (Freedom House)	41.59	24.50	54	2	94

**Table 2:** Pearson Correlation Coefficients

	GDP per capita	Percentage of Population under 14	Adult Unemployment rate	Percentage of Population over 25 years that have completed secondary education	Maternal Mortality Ratio	International Tourism, Number of Arrivals	Restri- ction on internal movements	International Travel Controls	Public Transport Closures	Freedom House Democracy Scores
GDP per capita	1.00									
Percentage of Population under 14	-0.80	1.00								
Adult Unemployment rate (Modeled ILO estimate)	0.48	-0.54	1.00							
Percentage of Population over 25 years that have completed secondary education	0.49	-0.45	0.31	1.00						
Maternal Mortality Ratio	-0.52	0.62	-0.36	-0.25	1.00					
International Tourism, Number of Arrivals	0.40	-0.46	0.46	0.72	-0.36	1.00				
Restriction on internal movements	0.25	-0.33	0.20	0.12	-0.13	0.30	1.00			
International Travel Controls	0.31	-0.08	0.00	-0.15	-0.16	-0.48	0.22	1.00		
Public Transport Closures	-0.06	-0.40	-0.05	0.13	-0.37	0.84	0.38	0.31	1.00	
Freedom House Democracy Scores	0.51	-0.54	0.33	0.14	-0.43	0.167	-0.12	-0.05	-0.20	1.00

**Table 3:** Results of Regression analysis for number of cases of COVID-19 per 100,000 population

Parameter	Estimate	Standard Error	t Value	Pr >|t|
**Intercept**	-5007.786658	3433.777208	-1.46	0.1577
**Region Central Africa**	1899.934314	696.406086	2.73	0.0117
**Region Eastern Africa**	439.486660	666.492030	0.66	0.5159
**Region Northern Africa**	860.482942	1154.375432	0.75	0.4633
**Region Southern Africa**	-132.822659	536.338759	-0.25	0.8065
**Region Western Africa**	0.000000			
**GDP per capita**	0.293836	0.159281	1.84	0.0774
**% pop. under 14 years**	88.981941	61.930246	1.44	0.1637
**Adult unemployment rate**	136.074484	35.719516	3.81	0.0009
**% pop.with secondary education**	-49.657597	17.206978	-2.89	0.0081
**Maternal Mortality Ratio**	-1.904539	0.983990	-1.94	0.0648
**International tourism numbers**	0.000074	0.000089	0.83	0.4129
**Public transport closure**	241.561694	344.017311	0.70	0.4893
**Internal Travel Restriction**	798.009176	233.889056	3.41	0.0023
**International Travel Restriction**	-427.063306	194.487422	-2.20	0.0380
**Freedom scores**	41.491799	14.389471	2.88	0.0082

**Table 4:** Results of Regression analysis for COVID-19 deaths per 100,000 population

Parameter	Estimate	Standard Error	t Value	Pr >|t|
**Intercept**	-123.7624041	78.18200917	-1.58	0.1265
**Region Central Africa**	40.2329925	15.85613269	2.54	0.0181
**Region Eastern Africa**	17.5188401	15.17503403	1.15	0.2597
**Region Northern Africa**	36.6062230	26.28341478	1.39	0.1765
**Region Southern Africa**	7.2255759	12.21163728	0.59	0.5596
**Region Western Africa**	0.0000000			
**GDP per capita**	0.0056725	0.00362658	1.56	0.1309
**% pop. under 14 years**	1.8953191	1.41005977	1.34	0.1915
**Adult unemployment rate**	2.9370346	0.81328035	3.61	0.0014
**% pop.with secondary education**	-1.5693819	0.39177733	-4.01	0.0005
**Maternal Mortality Ratio**	-0.0113024	0.02240400	-0.50	0.6185
**International tourism numbers**	0.0000070	0.00000202	3.48	0.0020
**Public transport closure**	5.2257070	7.83276345	0.67	0.5110
**Internal Travel Restriction**	8.9057363	5.32530657	1.67	0.1074
**International Travel Restriction**	-6.5629304	4.42818987	-1.48	0.1513
**Freedom scores**	0.8668921	0.32762689	2.65	0.0141

GDP, percentage of population under 14 years of age, Maternal Mortality Ratio, number of international tourists visiting per year and public transportation closures were not significant in predicting COVID-19 cases. Percentage of unemployed adults in the population, percentage of the population over 25 years of age with secondary education, Restriction on internal travel, International travel restrictions and level of democratization from Freedom House scores were all significantly associated with COVID-19 cases per 100,000 population. Higher unemployment results in higher COVID-19 rates while lower percentage of the population educated results in higher COVID-19 rates. Restrictions on internal movement results in higher COVID-19 rates. This was not the result we expected, and we had hypothesized that restrictions on internal movement would lead to less public crowding and less transmission of disease. It is possible however, that restriction of internal movement led to more indoor close contact interactions rather than outdoor interactions in public spaces which are less likely to result in COVID-19 transmission. Restrictions in international travel result in fewer COVID-19 cases and this is expected since cases of COVID generally came into African countries through people flying in from foreign countries, from countries outside the continent and putting international travel restrictions such as closing airports during the time under study which was if such actions were taken from the start of the pandemic till Dec 31^st^ 2020, would lead to having fewer cases. Higher levels of democratization results in more COVID-19 cases. Enforcing restrictions is easier in countries that are less free and less democratic, given that autocratically led countries can enforce draconian restrictions with little or no reaction from the population even if they are severely negatively impacted by such restrictions. In politically freer countries with higher levels of democracy, there is room for public debate and for parliaments to oppose or modify restrictions planned by government. For COVID-19 death, the regional difference is no longer significant, and unemployment rates and education were significant positively and negatively respectively associated, as they were for COVID-19 cases. Freedom scores was also significant as in with COVID cases, with higher level of democratization resulting in higher death rate. A variable that was not significant for COVID cases but was significant for COVID-19 deaths was the number of tourism visitors per year and none of the restriction variable for COVID-19 were significant in predicting COVID-19 death rate.

## Discussion

High unemployment, low education rates and higher democratization seem to be the drivers of COVID-19 rates in African countries in the first year of the pandemic. While advocating for lower unemployment and higher education is warranted not only for COVID-19 but also for all kinds of development reasons, advocating for less democratization for improvement in health outcomes is not. Most African autocratic leaders currently run competitive authoritarianism as defined by Levitsky and Way [17], in that they hold regular elections but make ensure the autocratic wins either by intimidation or violence against political opponents and their supporters. It can be argued that the short-term gain in health in such circumstances could not be sustained long-term based on the anarchy that envelopes the ends of dictatorships resulting from death, natural or otherwise of the autocrat or if the autocracy ends in a violent way. The reasons for higher unemployment and lower education rates leading to a better health outcomes are clear and recognized as pathways to improving human development overall [9]. Low education rates mean a significant part of the population will not be literate enough to understand public health communications and advisory messages and take adequate precaution even when messages are translated into local languages, this translation lags behind general daily news in the foreign national language. In addition, many languages do not have adequate translation for some to the scientific terms used in disease control and such translated information may not provide the same amount of understanding as when one understands the original language it was disbursed in. Harding [14], found democracy to be associated with better health outcomes for African countries in her analyses, she measured long-term health effects and more democracy probably improves health outcomes in the long-term but in pandemic situations where immediate actions need to be taken, dictators maybe more likely to be effective. Nega and Schneider [22] describe how the progress made by dictators in African countries end up resulting in backwash effects and thwarting the progress made. Progress in healthcare that is sustainable cannot come from autocratic rule but enforcing restrictive actions to thwart a pandemic may occur easier in an autocratic state than a democratic one.

That internal restrictions on movement had negative effect on COVID-19 rates for African countries is an important finding in our study. There was a lot of hardship experienced by people when the restrictions were imposed and because they may actually have enabled COVID-19 spread, it is important for countries to not use such restrictions in future pandemics especially in a pandemic were cases are coming in from international travel and not through local internal transmission with people especially poor people moving across borders. Internal restrictions disproportionally affected Africa’s poor economically while not aiding in limiting the spread of disease. A recent report on the effect of COVID-19 on employment around the world indicated that the Sub-Saharan Africa was second only to South-East Asia in lost pay and percentage of the population who stopped working during the early days of the pandemic [12]. This is of course due to the fact that these two regions have the least percentage of people whose jobs would allow them to work remotely. Such remote jobs would require having access to a computer and internet connection which are jobs available only to people already living above poverty levels. Sub-Saharan Africa had the highest rate for people who worked fewer hours due to COVID-19 again because the effect of limited internal movement will be more severe in the countries with less internet connections and ability to work from home. Therefore when a disease is most likely to be introduced through cases flying into the country, internal movements should be focused on such travelers and not the whole population, especially the poor, restrictions on people flying into the country and monitoring them for signs and symptoms would have caused less hardship to the poor and the economies in general. Restrictions imposed to limit disease spread should be based on the epidemiologic profile of each disease. Ebola restrictions in many countries warranted strict internal movement restrictions because the disease generally spread from rural areas to cities or vice-versa within countries and across land borders. Most countries did not acquire initial cases from infected persons flying and so infection control using restrictions on internal movements would work but for COVID-19 it may have resulted in people gathering indoors more rather than outdoors in public areas and expatiating spread. Restrictions on international travel was effective in reducing cases of COVID-19 and this alludes to the fact that cases generally came in through air flights. There was no effect of Public transportation closure and this is another restriction that created hardship for poorer members of the societies and did not have impact probably because the elite social class who travel internationally and could bring the disease back or associate with foreigners who also had potential to bring the disease into countries, do not generally use public transportation. Again a hardship was put in place which affected poorer members of society and had no benefit to them and may have been harmful. Ahmed et al. [2] found that COVID-19 lockdowns in African countries caused reduced access for other health issues in slum communities. Asongu et al. [3] also found that the enforcement of movement restrictions were counterproductive in African countries in their study of global WHO regions.

In our analyses GDP per capita was not associated with COVID-19 cases or death rate, given that COVID-19 reached African countries mostly through air flight it is comprehensible that the spread of the disease will be associated with higher GDP and ability of residents of a country to travel within their countries but we found no such association and country wealth did not determine cases or death rate. It seems receiving tourists in large numbers increased the exposure of countries to COVID-19 cases. The number of tourism data was for 2019 and predated the outbreak of the COVID-19 pandemic but it is expected that countries that have large number of tourist visitors would have continued having the high numbers until the pandemic was declared in March and countries started to implement restrictions and some tourists may have already brought in the disease before such actions were taken. Why number of tourism visitors would only be related to COVID-19 death rate and not rate of COVID-19 cases is unclear.

Leaders of African countries demonstrated strong leadership and collective action in their response to COVID-19 and this could help explain the relatively low number of cases compared to other regions of the world [4, 24] The fact that African countries are the least connected to international travel routes could also explain the limited numbers in the first year of the pandemic that we studied and the pattern of the disease spread could change as the pandemic as gone on. Currently African countries still have relatively low per capital cases and death, even as other regions which started with relatively low numbers such as India saw sharp increases in the early months of 2021. Early mathematical modelling indicated that swift action by African countries would be effective in limiting the the spread of COVID-19 in African countries [6, 20, 31]. Also, there have been extant reviews on what action should be taken to prevent widespread infection in African countries [10, 21]. The delay in the start of general population outbreaks in African countries due to its lower links to other regions most likely resulted in many countries already having preventive strategies in place when the disease started to spread. Also the first cases were amongst the elite who were the ones able to travel by flight and who are able to associate with visitors from countries where the disease was already widespread.

## Conclusion

In conclusion African countries should focus on improving education and reducing unemployment to better manage future epidemics. Shutting down tourism and international flights at the start of a pandemic would be reasonable if the pandemic started outside of the continent but probably difficult to achieve as countries that have high tourism rates depend on it economically. We chose variables that are available and may be important to the dynamics of the disease but also understand that some of these factors may not be practical to use for many countries with limited economies as occurs in most African countries.

For African countries, GDP per capita is not predictive of COVID-19 cases or death. The effect of countries wealth is independent of number of tourism visitors since many countries with tourism-based economies do not necessarily have high income. Our study also indicates that the countries with worst economies on the African continent are not the ones having the worst problems with COVID-19, in fact the reverse is true, and although not significant at *p* = 0.0774, there is a positive relationship between GDP and COVID cases. The main factors fueling the pandemic in African countries are high unemployment and low education, while autocracy enables lower rates and internal restrictions were not as effective but limiting international travel was.

## Data Availability

Spreadsheet with data used in analyses is available upon request and all data used is from publicly available sources as indicated in the text.

## Abbreviations

WHO: World Health Organization
GDP: Gross Domestic Product
HIV: Human Immunodeficiency Virus
AIDS: Acquired Immune Deficiency Syndrome

## References

[1] African Union Member States, Retrieved Aug 12, 2021, from https://au.int/en/member_states/countryprofiles2.

[2] Ahmed SA, Ajisola M, Azeem K, Bakibinga P, Chen Y-F, Choudhury NN, Fayehun O, Griffiths F, Harris B, Kibe P, Lilford RJ, Omigbodun A, Rizvi N, Sartori J, Smith S, Watson SI, Wilson R, Yeboah G, Aujla N (2020). Impact of the societal response to COVID-19 on access to healthcare for non-covid-19 health issues in slum communities of Bangladesh, Kenya, Nigeria and Pakistan: results of pre-COVID and covid-19 lockdown stakeholder engagements. BMJ Glob Health.

[3] Asongu SA, Diop S, Nnanna J (2020). The geography of the effectiveness and consequences of Covid-19 measures: global evidence. J Public Aff.

[4] Barnard H (2020). Another pandemic in Africa: weak healthcare, strong leadership, and collective action in Africa’s COVID-19 response. Manag Organ Rev.

[5] Bicalho C, Platas MR, Rosenzweig LR (2021). “if we move, it moves with us:” physical distancing in Africa during COVID-19. World Dev.

[6] Cabore JW, Karamagi HC, Kipruto H (2020). The potential effects of widespread community transmission of SARS-COV-2 infection in the World Health Organization African Region: a predictive model. BMJ Glob Health.

[7] Chang D, Chang X, He Y, Tan KJ (2022). The determinants of covid-19 morbidity and mortality across countries. Sci Rep.

[8] Chaudhry R, Dranitsaris G, Mubashir T, Bartoszko J, Riazi S (2020). A country level analysis measuring the impact of government actions, country preparedness and socioeconomic factors on COVID-19 mortality and related health outcomes. EClinicalMedicine.

[9] Deaton A (2003). Health, inequality, and economic development. J Econ Lit.

[10] Diop BZ, Ngom M, PouguéBiyong C, PouguéBiyong JN (2020). The relatively young and rural population may limit the spread and severity of COVID-19 in Africa: a modelling study. BMJ Glob Health.

[11] Freedom House 2021 democracy report. Retrieved Aug 20, 2021, from https://freedomhouse.org/countries/freedom-world/scores.

[12] Gallup. State of the Global Workplace 2021 Report. 2021.

[13] Haider N, Osman AY, Gadzekpo A, Akipede GO, Asogun D, Ansumana R, Lessells RJ, Khan P, Hamid MM, Yeboah-Manu D, Mboera L, Shayo EH, Mmbaga BT, Urassa M, Musoke D, Kapata N, Ferrand RA, Kapata P-C, Stigler F (2020). Lockdown measures in response to covid-19 in nine sub-saharan African countries. BMJ Glob Health.

[14] Harding R (2020). Who is democracy good for? elections, rural bias, and health and education outcomes in Sub-Saharan africa. J Polit.

[15] Jakhmola S, Baral B, Jha HC (2021). A comparative analysis of covid-19 outbreak on age groups and both the sexes of population from India and other countries. J Infect Dev Ctries.

[16] Lawal TA, Olapade-Olaopa EO (2017). Circumcision and its effects in Africa. Transl Androl Urol.

[17] Levitsky S, Way LA. Competitive authoritarianism: hybrid regimes after the cold war. Cambridge: Cambridge University Press; 2013.

[18] Liang L-L, Tseng C-H, Ho HJ, Wu C-Y (2020). Covid-19 mortality is negatively associated with test number and government effectiveness. Sci Rep.

[19] Mo P, Deng L, Liu X, Gao S, Liang K, Luo M (2020). Risk factors for delayed negative conversion of SARS-COV-2 in patients with covid-19 pneumonia: a retrospective cohort study. Epidemiol Infect.

[20] Mueller V, Sheriff G, Keeler C, Jehn M (2020). Covid -19 policy modeling in sub-saharan Africa. Appl Econ Perspect Policy.

[21] Musa HH, Musa TH, Musa IH (2021). Addressing africa’s pandemic puzzle: perspectives on covid-19 transmission and mortality in sub-saharan africa. Int J Infect Dis.

[22] Nega B, Schneider G (2012). Things fall apart: dictatorships, development, and democracy in Africa. J Econ Issues.

[23] Nolen S. Trying to solve a covid mystery: Africa’s low death rates. New York: New York Times; 2022.

[24] Obasanjo, I. 2021. Explaining the limited impact of COVID-19 in African countries. Afr Health J. https://africa-health.com/africa-health-journals/africa-health-july-2021-2-2-2-2-2.

[25] Our World in Data COVID-19, Retrieved Aug 12, 2021, from https://ourworldindata.org/policy-responses-covid.

[26] Oxford JS, Gill D (2018). Unanswered questions about the 1918 influenza pandemic: origin, pathology, and the virus itself. Lancet Infect Dis.

[27] Pengid S, Peltzer K, de Moura Villela EF, Fodjo JN, Siau CS, Chen WS (2022). Using Andersen’s model of health care utilization to assess factors associated with covid-19 testing among adults in nine low-and middle-income countries: an online survey. BMC Health Serv Res.

[28] Selvitella AM, Foster KL (2020). Societal and economic factors associated with covid-19 indicate that developing countries could suffer the most. Tech Soc Sci J.

[29] Silva J, Ribeiro-Alves M (2021). Social inequalities and the pandemic of covid-19: the case of rio de janeiro. J Epidemiol Community Health.

[30] Sorci G, Faivre B, Morand S. Explaining among-country variation in covid-19 case fatality rate. Sci Rep. 2020;10(1). 10.1038/s41598-020-75848-2. 10.1038/s41598-020-75848-2 PMC7609641 33144595

[31] van Zandvoort K, Jarvis CI, Pearson CA, et al. Response strategies for covid-19 epidemics in African settings: a mathematical modelling study. 2020. 10.1101/2020.04.27.20081711. 10.1186/s12916-020-01789-2 PMC7553800 33050951

[32] WHO COVID-19 pandemic declaration; 2020. https://www.who.int/director-general/speeches/detail/who-director-general-s-opening-remarks-at-the-media-briefing-on-covid-19---11-march-2020.

[33] WHO COVID-19 Dashboard WHO COVID-19 Dashboard. Retrieved Aug 12, 2021, from www.covid19.who.int/table.

[34] WHO Report of the WHO-China Joint Mission on Coronavirus Disease 2019 (COVID-19) 16–24 February 2020. https://reliefweb.int/report/china/report-who-china-joint-mission-coronavirus-disease-2019-covid-19?gclid=Cj0KCQjw8_qRBhCXARIsAE2AtRbtr_H-RoxPA3Blk4uLwV6qjTqtkQSv63LjEOmKAz3HhL4RUoy3WacaAsHIEALw_wcB.

[35] World Bank World Bank Data. Retrieved Aug 13, 2021, from https://data.worldbank.org/.

